# The Influence of Dopant Concentration on Optical-Electrical Features of Quantum Dot-Sensitized Solar Cell

**DOI:** 10.3390/molecules26102865

**Published:** 2021-05-12

**Authors:** Dang Huu Phuc, Ha Thanh Tung, Van-Cuong Nguyen, My Hanh Nguyen Thi

**Affiliations:** 1 Laboratory of Applied Physics, Advanced Institute of Materials Science, Ton Duc Thang University, Ho Chi Minh City 70880, Vietnam; danghuuphuc@tdtu.edu.vn; 2 Faculty of Applied Sciences, Ton Duc Thang University, Ho Chi Minh City 70880, Vietnam; 3 Faculty of Physics, Dong Thap University, Cao Lãnh 870000, Dong Thap Province, Vietnam; 4 Faculty of Chemical Engineering, Industrial University of Ho Chi Minh City, 12 Nguyen Van Bao, Go Vap, Ho Chi Minh City 70000, Vietnam; 5 Faculty of Mechanical Engineering, Industrial University of Ho Chi Minh City, 12 Nguyen Van Bao Street, Ward 4, Go Vap, Ho Chi Minh City 70000, Vietnam

**Keywords:** dopant concentration, quantum dots, cadmium selenite, cadmium sulfur

## Abstract

In this study, TiO_2_/CdS/Cd_x_Cu_1−x_Se, TiO_2_/CdS/Cd_x_Mn_1−x_Se, and TiO_2_/CdS/Cd_x_Ag_2−2x_Se thin films were synthesized by chemical bath deposition for the fabrication of photoanode in quantum-dot-sensitized solar cells. As a result, the structural properties of the thin films have been studied by X-ray diffraction, which confirmed the zinc Blende structure in the samples. The optical films were researched by their experimental absorption spectra with different doping concentrations. Those results were combined with the Tauc correlation to estimate the absorption density, the band gap energy, valence band and conduction band positions, steepness parameter, and electron–phonon interaction. Furthermore, the electrical features, electrochemical impedance spectrum and photocurrent density curves were carried out. The result was used to explain the enhancing performance efficiency.

## 1. Introduction

In recent years, nanocrystals have attracted attention from scientists globally due to their advanced features, such as high absorptivity, durable material composition, and efficient ruling of energies [1] Because of these properties, these semiconductors can be used in various fields, for example, as photoelectronics, photodetectors, quantum-dot-sensitized solar cell (QDSSCs), and other types of photoelectronic [2].

In recent decades, quantum dots (QDs) have attracted attention from scientists globally because of their unique properties, such as: high adsorption coefficient, stable chemical composition, and efficient control of the band gap (*E_g_*) [3,4]. Because of these properties, these semiconductors can be used in various fields, such as: photoelectronics, photodetectors, quantum-dot-sensitized solar cells, and other types of solar cells [5,6,7]. Over the years, CdSe QDs have been extensively investigated by scientists worldwide. They were applied to the photoanodes in the QDSSCs owing to their low cost, easy fabrication, and high stable chemical composition. However, the resistance of the CdSe films is significantly larger, and the energies of these films are smaller than those of bulk CdSe. Therefore, the amount of excited electrons generated in the conduction band (CB) of CdSe QDs to be transferred to the CB of TiO_2_ semiconductor was considerably low. Recently, a new technique has been applied to improve the current density and energy conversion of QDSSCs using CdSe QDs doped with transition metals such as Mg [5], and Co [8]. The exchange interaction between the transition metal and the electronic states of the CdSe QDs is fundamental for the study of optical and electrical properties of the QDSSCs. Generally, because of the doping of the transition metals in nano semiconductors, *E_g_* and high-absorbing photons are generated in different regions. As a result, the photocurrent density, which is due to the movement of the excited electrons in the circuit, increases [9,10]. Moreover, after doping, the recombination and dynamic resistance of TiO_2_/QDs and diffusion resistance of TiO_2_ film (R_ct2_), and counter electrode/electrolyte (R_ct1_) decrease significantly [11].

In previous articles, TiO_2_/CdS/Cd_x_Cu_1−x_Se (as 0%≤x≤5%, at%), TiO_2_/CdS/Cd_x_Mn_1−x_Se (as 0%≤x≤40%, at%), and TiO_2_/CdS/Cd_x_Ag_2−2x_Se 0%≤x≤40%, at% thin films have investigated and optimized characterizations of the materials as morphological analysis through FE-SEM, TEM; structure through XRD, Raman, EDX; optical properties as UV-Viss; lifetimes of excited electrons for fabricating photoanode in QDSSCs. In this research, we focus on the optical-electrical properties by their experimental absorption spectra with different doping concentrations and combination with the Tauc correlation to estimate the absorption density, the band gap energy, valence band and conduction band positions, steepness parameter, and electron–phonon interaction in the samples.

## 2. Results and Discussion

### 2.1. Structural Characterization

**Figure 1:**
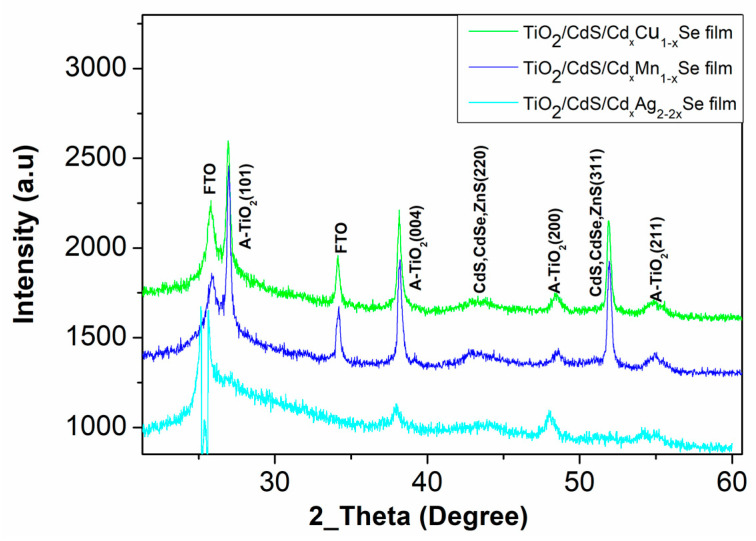
The X-ray diffraction of the TiO_2_/CdS/Cd_x_Cu_1−x_Se, and TiO_2_/CdS/Cd_x_Mn_1−x_Se and TiO_2_/CdS/Cd_x_Ag_2−2x_Se QDSSCs.

The structural TiO_2_/CdS/Cd_x_Cu_1−x_Se, TiO_2_/CdS/Cd_x_Mn_1−x_Se, and TiO_2_/CdS/Cd_x_Ag_2−2x_Se QDSSCs have been performed by X-ray diffraction. The obtained X-ray diffraction patterns are shown in Figure 1. For all thin films, the chart lists various parameters calculated from the diffraction peaks at 43.2° and 50.1° corresponding to the (220) and (311) crystal planes, respectively, of CdS, and CdSe zinc blende structure (JCPDS No. 88-2346 and JCPDS No. 41-1019) [12]. The peaks at 25.4°, 37.6°, 47.5° and 54.5° correspond to the (101), (004), (200), and (211) planes of the TiO_2_ Anatase structure [13]. In addition, the peak at 25 °C for TiO_2_/CdS/Cd_x_Cu_1−x_Se, TiO_2_/CdS/Cd_x_Mn_1−x_Se, and TiO_2_/CdS/Cd_x_Ag_2−2x_Se films conducted from the FTO, because all films were coated on the FTO [13].

### 2.2. Optical Characterization

**Figure 2:**
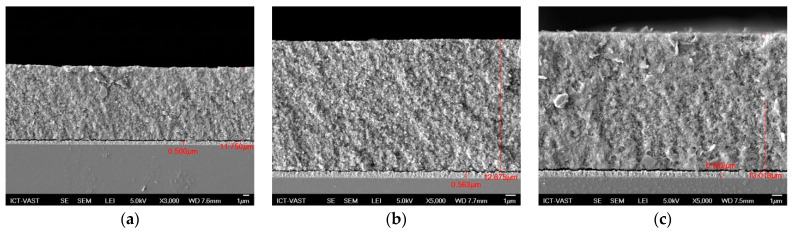
The FE-SEM of (**a**) TiO_2_/CdS/Cd_x_Cu_1−x_Se, (**b**) TiO_2_/CdS/Cd_x_Mn_1−x_Se, and (**c**) TiO_2_/CdS/Cd_x_Ag_2−2x_Se films.

In photovoltaic cells, both the optical and electrical features of component materials must be investigated extensively. In this study, the absorption spectra, transmission, and reflection spectra of QDSSCs were recorded using UV-Vis. From the absorption spectra, we can determine various optical parameters, such as the absorption coefficient, *E_g_* of materials to control further band tail and study the defect states inside materials. TiO_2_/CdS/Cd_x_Cu_1−x_Se, TiO_2_/CdS/Cd_x_Mn_1−x_Se, and TiO_2_/CdS/Cd_x_Ag_2−2x_Se films recorded approximately 11.7 µm, 12.675 µm, and 13.013 µm of thickness from Figure 2, respectively.

**Figure 3:**
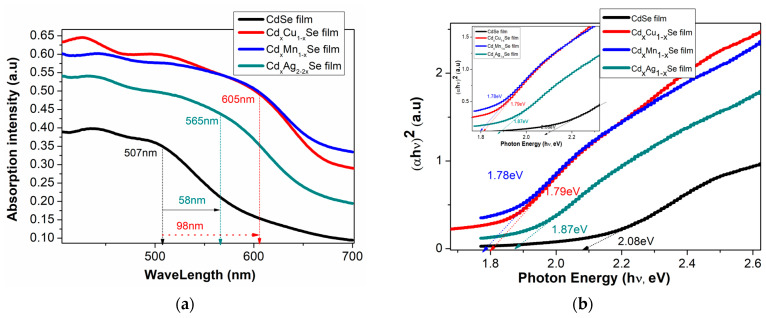

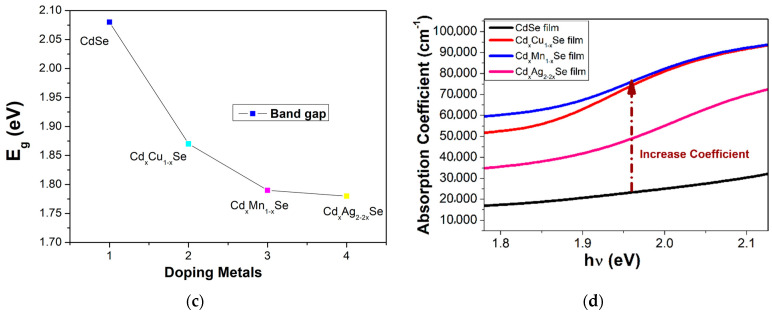
Variation in (**a**) the absorption spectra and (**b**) (*αhν*)^2^ plots with the changing Ag ratio and the dependence of (**c**) *E_g_* and (**d**) the absorption coefficient on the compositional TiO_2_/CdS/CdxCu_1−x_Se, and TiO_2_/CdS/Cd_x_Mn_1−x_Se, and TiO_2_/CdS/Cd_x_Ag_2-2x_Se thin films.

In Figure 3a, the absorbance density of the TiO_2_/CdS/Cd_x_Cu_1−x_Se, TiO_2_/CdS/Cd_x_Mn_1−x_Se, and TiO_2_/CdS/Cd_x_Ag_2−2x_Se thin films significantly increased by approximately 17%, when compared with undoped film with that of TiO_2_/CdS/Cd_x_Cu_1−x_Se photoanode. Moreover, the absorption density of the TiO_2_/CdS/Cd_x_Ag_2−2x_Se thin films decreased by approximately 11%, when compared with TiO_2_/CdS/Cd_x_Cu_1−x_Se thin films. Furthermore, the absorption density of TiO_2_/CdS/Cd_x_Cu_1−x_Se, TiO_2_/CdS/Cd_x_Mn_1−x_Se, and TiO_2_/CdS/Cd_x_Ag_2−2x_Se photoanodes shifted towards a longer wavelength (from 507 nm for undoped films to 605 nm for doped films). This is attributed to the replacement of the Cd atoms by the metal atoms in the films and a proper crystallization of the films corresponding to a suitable doping-concentration-reducing effect state [14]. The 605 nm absorption peak of the TiO_2_/CdS/Cd_x_Cu_1−x_Se film was attributed to the transition of the *VB* of Se to d-state of Cu dopant in the *E_g_* of pure CdSe QDs [15].

From the UV-Vis spectra, using the Tauc relation in Equation (1), we variously determined the *E_g_* of the TiO_2_/CdS/Cd_x_Cu_1−x_Se, TiO_2_/CdS/Cd_x_Mn_1−x_Se, and TiO_2_/CdS/Cd_x_Ag_2−2x_Se films with dopants [16]

(1) $$\alpha h\nu =\alpha_{o}{\left(h\nu -E_{g}\right)}^{n}$$

where *α* is noted as a coefficient of light coming adsorption, hν is noted as a light coming energy, *α_o_* is not the change factor, CdSe QDs has the direct band gap, and *n* = 0.5. After plotting Equation (1) in Figure 3b, *E_g_* of the films was estimated and recorded (shown in Table 1).

Figure 3b,c illustrates the valuable *E_g_* for CdSe-doped photoanodes varying from 1.87 eV to 1.78 eV, which is smaller than that without doped photoanode (2.08 eV). Furthermore, by observation, the *E_g_* values of CdSe-doped photoanodes depended significantly on the change in dopants. Here, the reduced *E_g_* of the doped films resulted from two factors: an increase in particle size after doping [17], and a change in force density on account of the presence of Cu-Se, Mn-Se, and Ag-Se pairs in the extended lattice at the expense of Cd-Se in the host lattice [18]. Therefore, the dopant energy levels, called transition energies, are presented in the *E_g_* of pure CdSe QDs. Subsequently, these photons obtain energy below *E_g_* of pure CdSe nano semiconductor, which can be absorbed at dopant energy levels before they are transferred to the *CB* of pure CdSe semiconductor. Therefore, J_SC_ was significantly increased on account of a large number of absorbed photons and, consequently, the absorption peak shifted towards to a longer wavelength. Furthermore, both the conduction band and valence band positions of the TiO_2_/CdS/Cd_x_Cu_1−x_Se, TiO_2_/CdS/Cd_x_Mn_1−x_Se, and TiO_2_/CdS/Cd_x_Ag_2−2x_Se films were estimated by the Tauc formula and listed in Table 1. Obtained *CB* values of −3.98, −3.7, and −4.1 eV corresponding to the TiO_2_/CdS/Cd_x_Cu_1−x_Se, TiO_2_/CdS/Cd_x_Mn_1−x_Se, and TiO_2_/CdS/Cd_x_Ag_2−2x_Se films, respectively, are higher than the *CB* value of −4.105 eV of pure CdSe QDs. Therefore, the presence of doping metals, which has increased the *CB* value in the doped films compared to that in the undoped CdSe QDs, aids in the transition of excited electrons from the CdSe QDs to TiO_2_.

Due to the band structure and transition energies of materials in the absorption process, the absorption coefficient is estimated using an equation correlation [19], as follows

(2) $$\alpha =\frac{2.3026.A}{d}$$

where *A* and *d* are the absorption density and the thickness of the film, respectively.

As can be seen in Figure 3d, α significantly increases after doping, and a shift in the absorption peak for CdSe doping metals compared to that without dopant occurs. This result, obtained from the replacement of Cd atoms by the metal dopants in the doped films, which induces the d-state electrons of dopant atoms, appeared in *E_g_* of CdSe QDs to introduce the energy levels of dopant in the *E_g_* of CdSe QDs. Therefore, the absorption coefficient of the doped films was enhanced significantly because the appearance of the transition energy levels ensured that a large number of photons was absorbed in different regions [20,21].

To correlate the localized states with the *E_g_* of the films, the Urbach energy (*E_u_*) of the films was determined as follows [22]

(3) $$\alpha =\alpha_{o}\exp\left(\frac{h\nu }{E_{u}}\right)$$

**Figure 4:**
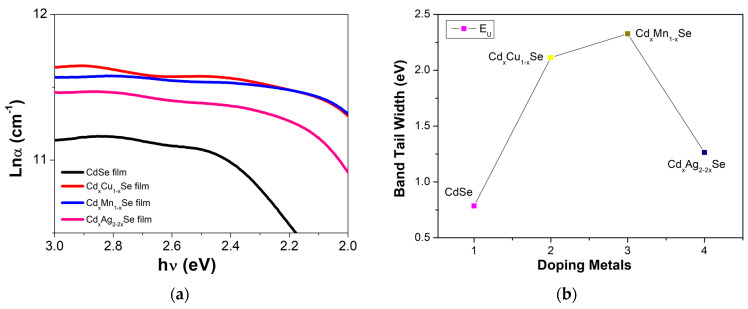
The dependence of (**a**) the ln(α) of the films and (**b**) the Band tail energy on the compositional TiO_2_/CdS/CdxCu_1−x_Se, and TiO_2_/CdS/Cd_x_Mn_1−x_Se and TiO_2_/CdS/Cd_x_Ag_2−2x_Se thin films.

The Urbach plots between ln(*α*) and hν and *E_u_* (which are estimated from the slope of the curves between ln(*α*) and hν) are shown in Figure 4. As is evident, the valuable *E_u_* depends on the type of doping metal. There was a minor decrease in its *E_u_*, from 0.786 eV (undoped film) to 2.33 eV (doped films), due to the reduced defect states in the TiO_2_/CdS/Cd_x_Cu_1−x_Se, TiO_2_/CdS/Cd_x_Mn_1−x_Se, and TiO_2_/CdS/Cd_x_Ag_2−2x_Se films [23]. The *E_u_* value of 2.33 eV was achieved for Cu doping, and was approximately two times higher than the band tail energy values for Mn and Ag doping (shown in Table 1). This implies that the large defect states in the films are a result of the unsatisfied bond, and the center of those defect states in *E_g_* of the TiO_2_/CdS/Cd_x_Cu_1−x_Se, TiO_2_/CdS/Cd_x_Mn_1−x_Se, and TiO_2_/CdS/Cd_x_Ag_2−2x_Se films. Depending on the dopant, the defect state center can appear in the middle of *E_g_*, near the *VB*, or near the *CB* [24].

**Figure 5:**
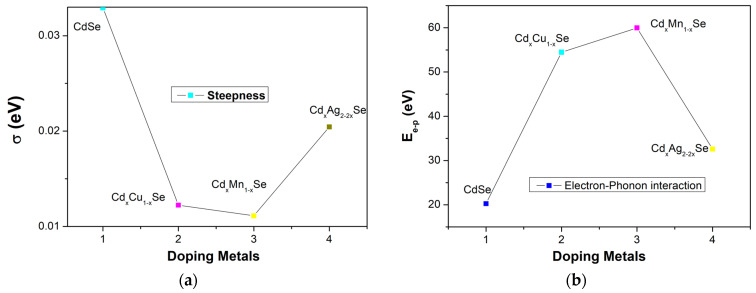
The dependence of (**a**) *σ* and (**b**) *E*_e-p_ in metal addition in films.

The absorption spectrum of the photovoltaic cells depends on several factors, such as the type of doping material, doping concentration, photoconductivity, thickness of the film, and especially, photon absorption in film. The steepness parameter, σ, is considered to result from the electron–phonon interaction (*E_e-p_*). σ is correlated with *E_e-p_* via the following correlation: *E_e-p_* = 3/2σ. Figure 5 and Table 1 show the variations in σ and *E_e-p_*, respectively. As can be seen in Figure 4a, σ shows a downward trend as the concentration of the doping metal reduced to a minimum of 3% (0.011 eV), which implies a stronger electron–phonon interaction energy corresponding to the TiO_2_/CdS/Cd_x_Cu_1−x_Se film as a result, confirming that the bond energy is as a parameter of film composition [24]. Furthermore, the *E_e-p_* value for the TiO_2_/CdS/Cd_x_Cu_1−x_Se film (59.97 eV) is significantly higher than for the TiO_2_/CdS/Cd_x_Ag_2−2x_Se film (32.6 eV) or the CdSe film (20.25 eV), and σ increases considerably. Evidently, the compositional variations in ternary TiO_2_/CdS/Cd_x_Cu_1−x_Se, TiO_2_/CdS/Cd_x_Mn_1−x_Se, and TiO_2_/CdS/Cd_x_Ag_2−2x_Se thin films caused variations in the optical parameters [24].

### 2.3. Electrical Properties

**Figure 6:**
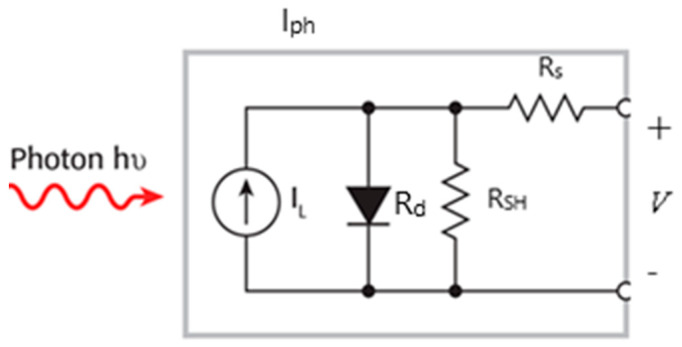
The circuit of a photovoltaic cell [25].

In this section, we study the current–voltage (J-V) characteristics of QDSSCs and, subsequently, the estimated primary physical parameters using one illuminated J-V curve. The photovoltaic device significantly depends on the four resistances in a photovoltaic cell: an external resistance (*R_D_*), internal resistance (*R_d_*), series resistance (*R_s_*), and a shunt resistance (*R_SH_*) (Figure 6). Ideally, R_s_ should be zero, and *R_SH_* should be infinite. As a result of the dynamic parameters, significant power losses caused by the dynamic resistances due to various factors, such as manufacturing defects, technological processes, and natural materials, are estimated.

In [26], we conducted the equations for dynamic parameters as follows

(4) $$R_{D}=\frac{V_{1}-V_{2}}{I_{2}-I_{1}}$$

(5) $$R_{d}=\frac{1}{\alpha \left(I_{2}-I_{1}\right)}Ln\left(\frac{I_{ph}+I_{o}-I_{1}}{I_{ph}+I_{o}-I_{2}}\right)\;$$

(6) $$R_{s}=R_{D}-R_{d}$$

(7) $$R_{SH}=\frac{V_{OC}}{I_{ph}-I_{o}\left(e^{\alpha V_{OC}}-1\right)}$$

(8) $$\alpha =\frac{q}{nk_{B}T}$$

where *q* and *k_B_* are the electronic charge and Boltzmann constant, respectively, *T* is the room temperature, *I_ph_* is the incident light intensity, and *I_o_* is the diode saturation current. The obtained physical parameters are listed in Table 2.

**Figure 7:**
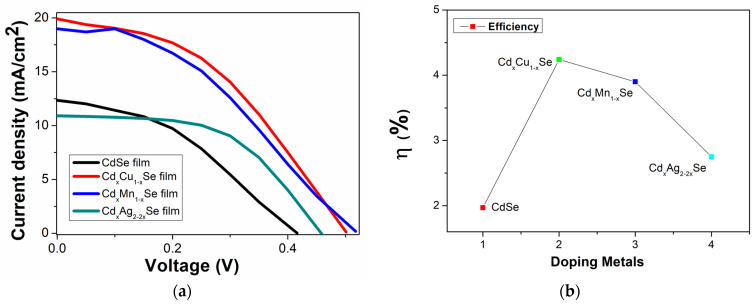
(**a**) Variation of photocurrent density–voltage curves and (**b**) dependence of the performance efficiency on metal addition in films.

In this experiment, samples of photovoltaic cells fabricated using the TiO_2_/CdS/Cd_x_Cu_1−x_Se, TiO_2_/CdS/Cd_x_Mn_1−x_Se, and TiO_2_/CdS/Cd_x_Ag_2−2x_Se photoanodes were recorded by a Keithley 2400 source meter under identical illumination condition. Figure 7a,b show the graphical representation of the measurement results. The performance efficiency of the photovoltaic cells is sensitive to the concentration of the metal dopants (Figure 6). For example, the energy conversion value increased significantly from 1.97% (undoped film) to 4.24% (TiO_2_/CdS/Cd_x_Cu_1−x_Se film), and the highest efficiency was obtained for Cu doping. Furthermore, the efficiency dropped to 2.75% (for TiO_2_/CdS/Cd_x_Ag_2−2x_Se film). Furthermore, the valuation parameters of the photovoltaic cells were significantly influenced by doping metals; the corresponding experimental values are recorded in Table 2.

**Figure 8:**
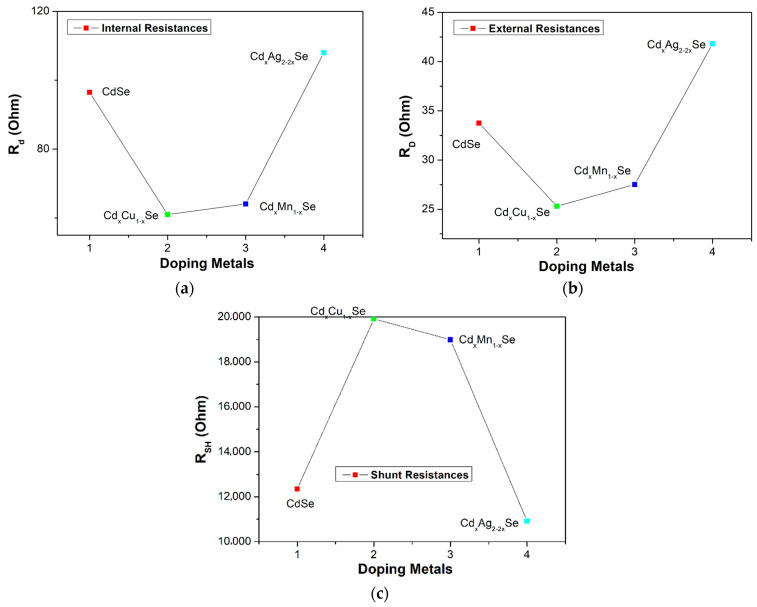
Dependence of (**a**) the external resistance, (**b**) internal resistance, and (**c**) shunt resistance on metal addition in films.

The values of the *R_D_*, *R_d_*, and *R_SH_* of the photovoltaic cells are extracted from the photocurrent density curves in Figure 8a–c, and one J-V curve method, and listed in Table 2. Moreover, *R_s_* is the resistance between the external circuit and the counter electrode photoanode. Generally, *R_s_* drops rapidly with the doping of metal dopants because of the difference between *R_D_* and *R_d_* value of *R_s_* decreased from 62.8 Ω (undoped film) to 35.7 Ω (TiO_2_/CdS/Cd_x_Cu_1−x_Se film). Here, *R_D_* is the resultant of *R_SH_*, *R_d_* (through the diode), and *R_s_*. The value of *R_D_* was lowest for the TiO_2_/CdS/Cd_x_Cu_1−x_Se film, and increased to attain 66.2 Ω for the TiO_2_/CdS/Cd_x_Ag_2−2x_Se film. Generally, the value of *R_D_* of the doped films is lower than that of the undoped film because the replacement of the Cd atoms in the CdSe film hosted by the metal dopants reduced resistance inside each layer and at multilayer surfaces. Moreover, a leakage current generated in the technology process can change *R_D_*. Therefore, while *R_SH_* should be infinite for an ideal photovoltaic cell, practically, there is an upper limit of 20 kΩ for *R_SH_*. According to Table 2, the *R_SH_* values for doped films are higher without dopant, and the highest, 19.9 kΩ, is obtained from the TiO_2_/CdS/Cd_x_Cu_1−x_Se film. Evidently, *R_SH_* is sensitive to the doping metal; while, for C doping, *R_SH_* reaches a maximum value of 19.9 kΩ, for Ag doping, *R_SH_* value decreased to 10.92 kΩ for TiO_2_/CdS/Cd_x_Ag_2−2x_Se film. This result was also proven as the UV-Vis peak moves to a longer wavelength owing to the reduced *E_g_* and enhanced *E_e-p_* discussed in Section 3.2. Furthermore, our result also agrees with reference [26].

Conventionally, electrochemical impedance spectroscopy (EIS) was found by Mosa–Sero [27] to determine the occurring processes through contacts, TiO_2_ film and polysulfide electrolyte. These investigation and analysis processes included several steps, such as the pumping of excited charges from the *CB* of QDs to the *CB* of TiO_2_, diffusive charges inside the TiO_2_ film before moving to the external circuit, the transfer of electrons to the counter electrode/polysulfide. In this case, an equivalent circuit was extracted from the EIS fitting using the Nova software. As far as the EIS fitting is concerned, the equivalent circuits for simulation were chosen for a consistent match between the EIS experiment and EIS fitting. Subsequently, *R_ct1_* and *R_ct2_* were obtained from EIS fitting. Those values are listed in Table 2.

**Figure 9:**
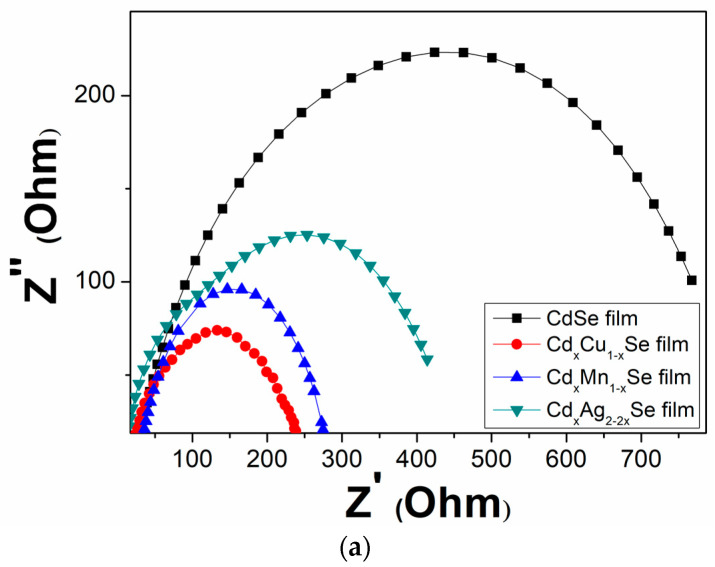

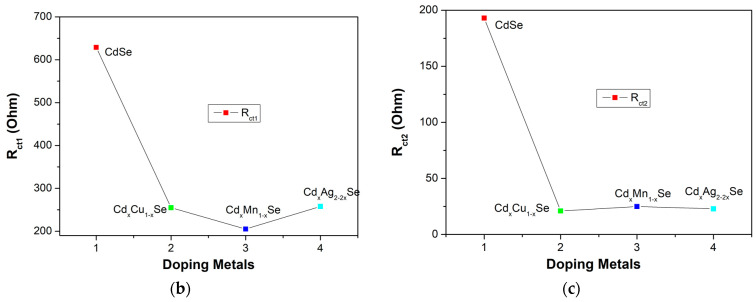
(**a**) Variation of the Electrochemical Impedance Spectra and (**b**) dependence of the recombination resistances: (**b**) polyelectrolyte and polyelectrolyte/counter electrode contact (*R_ct1_*) and (**c**) TiO_2_/QDs contact and diffusion resistance in TiO_2_ films (*R_ct2_*).

Figure 9a–c shows the EIS curves of the photovoltaic cells based on the metal dopants, and dynamic resistance curves in the counter electrode/polysulfide electrolyte surface, and in the redox process of electrolysis, *R_ct1_*, and the recombination resistance at TiO_2_/QDs surface, and in TiO_2_ film, *R_ct2_*. In the same way, the recombination resistances, which are listed in Table 2, depend significantly on the doping concentration of the metal dopants. A downward trend was observed *R_ct1_* value from 629 Ω (undoped film) to 205 Ω (TiO_2_/CdS/Cd_x_Mn_1−x_Se film) or to 255 Ω (TiO_2_/CdS/Cd_x_Cu_1−x_Se film). The value of *R_ct1_* was the lowest (205 Ω) for the TiO_2_/CdS/Cd_x_Mn_1−x_Se film, and it increased to a maximum value of 256Ω for the TiO_2_/CdS/Cd_x_Ag_2-2x_Se film. This implies that a decrease in the recombination resistance at the counter electrode/polysulfide electrolyte surface and in the redox process of the electrolyte resulted in the enhanced current density (19.89 mA/cm^2^) and energy conversion (4.24%) compared to those for the undoped film (12.4 mA/cm^2^ and 1.97%, respectively) [28]. Furthermore, as mentioned above, the correlation with *R_ct2_* caused the recombination resistance in the photovoltaic cells, which indicates that the recombination processes at TiO_2_/QDs surface, inside QDs, and the diffusion of charges in TiO_2_ film were correlated to the TiO_2_/CdS/Cd_x_Cu_1−x_Se film, TiO_2_/CdS/Cd_x_Mn_1−x_Se film, and TiO_2_/CdS/Cd_x_Ag_2-2x_Se film. Therefore, *R_ct2_* is pivotal in explaining the enhanced current density and performance efficiency after the proposed metal doping. Correspondingly, the values of the *R_ct2_* decreased from 193 Ω (undoped film) to 21 Ω (Cu doping). The value of *R_ct2_* was the lowest for Cu doping (21Ω), and increased to a maximum of 23 Ω for Ag doping. This result is consistent with the results from the photocurrent density curve, absorption spectra, and the band tail energy. Because of the reduced recombination resistance, a large number of charges accumulated, further reducing the recombination process of the excited electrons with the surface states of QDs, resulting in the fast pumping of excited charges between CdS, CdSe and TiO_2_ [29].

## 3. Experiment

### 3.1. Materials

TEC7 Glass Plates 3.2 mm (2.2 mm of thickness, 1.2 × 2 cm = 0.196 cm^2^ of area) was purchased from Sigma in Frankfurter, German. Titania paste, Sodium Selenite (Na_2_SeO_3_), Cd(NO_3_)_2_. 2H_2_O, Na_2_S.9H_2_O, Cu(NO_3_)_2_. 3H_2_O, Mn(CH_3_COO)_2_.4H_2_O, AgNO_3_, Na_2_SO_3_, NaOH, Se powder, KCl, Na_2_S, S were bought from Sigma in Frankfurter, German. All chemicals were used directly without further purification.

### 3.2. Preparation Processes

TiO_2_ film was coated on the Fluorine-doped tin oxide (FTO) followed by annealing in air at 500 °C.

Fabrication of CdS film: The FTO/TiO_2_ film was dipped in Cd^2+^-solution (2.665 g Cd(NO_3_)_2_.2H_2_O dissolved in a 20 mL mixture of ethanol and pure water in 1:1 ratio) for 5 min, and then washed with ethanol. Furthermore, it was dipped in S^2−^ solution (12 g of Na_2_S.9H_2_O, dissolved in 100 mL mixture of methanol and pure water in 1:1 ratio) for 5 min and then washed with methanol. This two-step process formed a single layer of the CdS film. Two layers of the CdS film were coated by repeating the two-step process.

#### Fabrication of Cu^2+^ Doped, Mn^2+^ Doped, and Ag^+^ Doped CdSe Films

For Cu^2+^ doping, Cd^2+^ and Cu^2+^-solution (0.1 M Cd(NO_3_)_2_.2H_2_O and 0.1 M Cu(NO_3_)_2_.3H_2_O dissolved in a 30 mL mixture of ethanol and pure water in 1:1 ratio) was kept for 5 min, and then washed with ethanol.

For Mn^2+^ doping, Cd^2+^ and Mn^2+^-solution (0.1 M Cd(NO_3_)_2_. 2H_2_O and 0.1 M Mn(CH_3_COO)_2_. 4H_2_O dissolved in 30 mL mixture of methanol and pure water in 1:1 ratio) was kept for 5 min, and then washed with methanol.

For Ag^+^ doping, Cd^2+^ and Ag^+^-solution (0.1 M Cd(NO_3_)_2_. 2H_2_O and 0.1 M AgNO_3_ dissolved in a 30 mL mixture of ethanol and pure water in 1:1 ratio) was kept for 5 min, and then washed with ethanol.

Furthermore, for the fabrication of Cu^2+^, Mn^2+^, and Ag^+^ doped films, the previously synthesized FTO/TiO_2_/CdS were, respectively, separately dipped into the solutions mentioned above. Subsequently, the films were dipped in Se^2−^-solution (2.27 g Se powder and 0.6 M Na_2_SO_3_ were dissolved in 100 mL of water and then added to 5 mL of 1 M NaOH. Furthermore, the solution was stored at 70 °C in 7 h) for 15 min at 50 °C, and then washed with pure water to form a single layer of the CdSe film desired doping material. Three layers of the CdSe film with the desired doping material were coated by repeating the above process. Finally, the films were annealed in the range from 120 °C to 300 °C as in vacuum for 30 min.

The polysulfide electrolyte was prepared by mixing 0.5 M Na_2_S, 0.2 M S, and 0.2 M KCl in pure water in 7:3 ratio.

The photoanodes have been synthesized and characterized using XRD (Philips, PANalytical X’Pert, CuKα radiation). The absorption spectra were recorded using a UV-Vis JASCO V-670. The completed photovoltaic cells were characterized using a Keithley 2400 source meter developed by SOLARENA, Sweden. The obtained J-V curves are supported to estimate the valuable electrical range of devices. The scanning speed voltage of the equipment was set at 5 mV/s in the range from V_OC_ to 0. The electrochemical impedance spectroscopy was performed using an impedance analyzer (ZAHNER CIMPS).

## 4. Conclusions

TiO_2_/CdS/Cd_x_Cu_1−x_Se, TiO_2_/CdS/Cd_x_Mn_1−x_Se, and TiO_2_/CdS/Cd_x_Ag_2-2x_Se thin films were successfully synthesized on FTO glass as photoanodes in solar cells, which were sensitive to Cu_2_S counter electrode. The QDSSCs using the TiO_2_/CdS/Cd_x_Cu_1−x_Se photoanode exhibits the highest performance: *J*_SC_ = 19.89 mA/cm^2^, *V*_OC_ = 0.505 V, *FF* = 0.379, and *η* = 4.24% as a result of enhancing electrocatalytic activity, charge transfer and charge collection, reducing recombination, and the longer excited electron lifetime. The optical parameters also are sensitive to dopant. This implies that large defect states in the films are a result of the unsatisfied bond and the center of those defect states in *E_g_* of Cd_x_Cu_1−x_Se films. They can be seen in the middle of *E_g_*, near the *VB*, or near the *CB*. Furthermore, the electrical properties of the Cd_x_Cu_1−x_Se were also investigated through the *R_s_*, *R_SH_* dynamic resistance, recombination resistance, diffusion resistance in TiO_2_ layer, and TiO_2_/QDs, *R_ct1_*, *R_ct2_*. As a result, the recombination and dynamic resistance of TiO_2_/QDs and diffusion in TiO_2_ (*R_ct2_*) films, and the diffusion at the counter electrode/electrolyte (*R_ct1_*), decreased significantly.

## Figures and Tables

**Table 1:** *E_g_*, *CB*, and *VB* were estimated by Tauc equation and the band tail, *σ*, *E_e-p_* were obtained from the absorption spectra.

Atom%	*E_g_* (eV)	*X* (eV)	*E_CB_* (eV)	*E_VB_* (eV)	*E_U_* (eV)	*σ* (eV)	*E_e-p_* (eV)
CdSe film	2.08	5.145	−4.105	−6.2	0.786	0.033	20.25
Cd_x_Cu_1−x_Se film	1.87	4.91	−3.98	−5.85	2.11	0.012	54.5
Cd_x_Mn_1−x_Se film	1.79	4.62	−3.7	−5.5	2.33	0.011	59.97
Cd_x_Ag_2−2x_Se film	1.78	4.99	−4.1	−5.88	1.26	0.0205	32.6

**Table 2:** The values of the performance efficiency (efficiency, short-circuit current, open-circuit voltage, fill factor) and *R_D_*, *R_d_*, *R_SH_*, *R_s_* were estimated from J-V curves and *R_ct1_*, *R_ct2_* were obtained from the EIS experiment.

Atom%	*J*_SC_ (mA/cm^2^)	FF	*V_OC_* (V)	η (%)	*R_D_* (Ω)	*R_d_* (Ω)	*R_SH_* (kΩ)	*R_S_* (Ω)	*R_ct1_* (Ω)	*R_ct2_* (Ω)
CdSe film	12.4	0.379	0.42	1.97 ± 0.01	33.75	96.5	12.4	62.8	629	193
Cd_x_Cu_1−x_Se film	19.89	0.419	0.505	4.24 ± 0.003	25.31	61	19.9	35.7	255	21
Cd_x_Mn_1−x_Se film	18.99	0.381	0.52	3.9 ± 0.005	27.51	64.1	18.99	36.6	205	25
Cd_x_Ag_2−2x_Se film	11	0.53	0.47	2.75 ± 0.008	41.81	108	10.92	66.2	256	23

## Data Availability

The data presented in this study are available on request from the corresponding author.
